# Performance Evaluation of Tight Ultrafiltration Membrane Systems at Pilot Scale for Agave Fructans Fractionation and Purification

**DOI:** 10.3390/membranes10100261

**Published:** 2020-09-27

**Authors:** Noe Luiz-Santos, Rogelio Prado-Ramírez, Enrique Arriola-Guevara, Rosa-María Camacho-Ruiz, Lorena Moreno-Vilet

**Affiliations:** 1 Centro de Investigación y Asistencia en Tecnología y Diseño del Estado de Jalisco A.C. Camino arenero 1227. El Bajío, C.P. 45019 Zapopan, Jalisco, Mexico; noeibq24@gmail.com (N.L.-S.); rcamacho@ciatej.mx (R.-M.C.R.); 2 Departamento de Ingeniería Química, CUCEI-Universidad de Guadalajara, Blvd. M. García Barragán 1421, C.P. 44430 Guadalajara, Jalisco, Mexico; arriole@hotmail.com; 3 CONACYT- Centro de Investigación y Asistencia en Tecnología y Diseño del Estado de Jalisco A.C. Av. Normalistas 800, Colinas de la Normal, C.P. 44270 Guadalajara, Jalisco, Mexico

**Keywords:** fine ultrafiltration, agave fructans, ceramic membrane, polymeric membrane, rejection coefficient

## Abstract

Ceramic and polymeric membrane systems were compared at the pilot scale for separating agave fructans into different molecular weight fractions that help to diversify them into more specific industrial applications. The effect of the transmembrane pressure of ultrafiltration performance was evaluated through hydraulic permeability, permeate flux and rejection coefficients, using the same operating conditions such as temperature, feed concentration and the molecular weight cut-off (MWCO) of membranes. The fouling phenomenon and the global yield of the process were evaluated in concentration mode. A size distribution analysis of agave fructans is presented and grouped by molecular weight in different fractions. Great differences were found between both systems, since rejection coefficients of 68.6% and 100% for fructans with degrees of polymerization (DP) > 10, 36.3% and 99.3% for fructooligosaccharides (FOS) and 21.4% and 34.2% for mono-disaccharides were obtained for ceramic and polymeric membrane systems, respectively. Thus, ceramic membranes are better for use in the fractionation process since they reached a purity of 42.2% of FOS with a yield of 40.1% in the permeate and 78.23% for fructans with DP > 10 and a yield of 70% in the retentate. Polymeric membranes make for an efficient fructan purification process, eliminating only mono-disaccharides, and reaching a 97.7% purity (considering both fructan fractions) with a yield of 64.3% in the retentate.

## 1. Introduction

Native agave fructans are a heterogeneous mixture of branched fructose polymers, linked by glycosidic linkages of fructose–fructose β (2−1) and β (2−6), with intermediate or terminal glucose units with degrees of polymerization (DP) between three and 29 [1]. These fructans have potential applications due to their proven beneficial effects on human health, such as the prebiotic effect, as well as decreasing the body mass index, total body fat and triglyceride levels [2,3,4,5] and their technological applications as encapsulating agents and as substitutes for fat in food [6,7,8,9,10]. However, it has been reported that fructans with different DP differ in their prebiotic effectiveness and techno-functional properties, as well as in the removal of low-molecular weight sugars such as glucose, fructose and sucrose, increasing the purity and functionality of fructans. For example, the fraction of agave fructans with DP ˃10 improves the biological effect of triglyceride reduction in relation to native fructans, while the enriched fraction of fructooligosaccharides (FOS) enhances the decrease in body weight, body fat, hyperglycemia and hepatic steatosis [11,12,13]. For these reasons, the membrane process has been proposed to obtain agave fructans with a higher purity [14,15,16] and to obtain different DP fractions [17,18].

The ultrafiltration (UF) process uses a membrane as a selective barrier according to its molecular weight cut-off (MWCO) in the range from 1 to 300 kDa [19], where the sieving effect dominates the UF process when using membranes with an MWCO greater than 4 kDa. However, separation using tight UF membranes (from 1 to 3 kDa) becomes more complex because it combines the sieving effect and Donnan exclusion, like the phenomenon that dominates the nanofiltration process (from 200 Da to 1 kDa). In some cases, the membrane with 1 kDa MWCO is considered a nanofiltration membrane because it is at the cut-off limit and is used for decolorization, phosphate elimination, and the purification of oligosaccharides [14,20,21,22].

The information given by the suppliers is limited to the MWCO, which is defined as the molecular weight (MW) at which 90% of the solute is rejected by the membrane; however, there is no standardized procedure for this test, so there could be variability under different conditions [23]. Furthermore, rejection is affected by the molecular shape of the solute, the membrane–solute interaction, the configuration of the membrane, and the interaction between the solutes and the concentration polarization phenomenon, which can reduce the size of the pores and affect the separation [24,25]. 

In addition to the above, choosing the membrane configuration and its material is crucial for each application, since it depends on obtaining good separation and yields. Currently, polymeric membranes have a greater application in the industry; however, ceramic membranes have gained interest in recent years. It is important to realize that each system has advantages and disadvantages, depending on the application. In this sense, polymeric membranes are more sensitive to temperature and pH conditions in cleaning cycles. Proper cleaning and maintenance can allow polymer membranes to be replaced every 1.5 years, while ceramic membranes can have a lifespan of approximately 7 years [26]. From the economic–commercial perspective, the cost of installing a ceramic membrane is 80–90% higher than that corresponding to a polymeric membrane, while, in terms of membrane prices, the ratio is approximately 4:1 [27,28]. It is important to mention that membrane fouling is of great concern for the application of membrane technology [29,30,31,32,33,34], which is reflected in the flux decrease resulting from the clogging of the membrane pore, which can be an irreversible phenomenon in some cases. The information generated in the experimental evaluation is necessary for the implementation of a successful separation process [28].

Thus, in order to choose the right system, some authors have studied the comparison between ceramic and polymeric membranes for different purposes and applications [35,36,37,38]. Few studies have been carried out using the same membrane cut-off and the same operating conditions [39,40] and none have been applied to agave fructan fractionation. The industry must define the purification and fractionation process for agave fructans and, for these reasons, the objective of this study was to comparatively evaluate the operation of ceramic and polymeric membrane systems at the pilot scale in the tight UF process of agave fructans, in terms of their hydraulic permeability, permeate flux, rejection coefficient, fouling resistance and the global yield of the process.

## 2. Materials and Methods

### 2.1. Agave Fructans

The aqueous solution of 10 kg·m^−3^ *Agave tequilana* fructans (Olifructine^®^) was prepared from syrup at 70 kg·m^−3^, which was kindly provided by Nutriagaves de Mexico (Jalisco, Ayotlan, Mexico).

### 2.2. Pilot Scale Filtration System and Membranes

**Figure 1:**
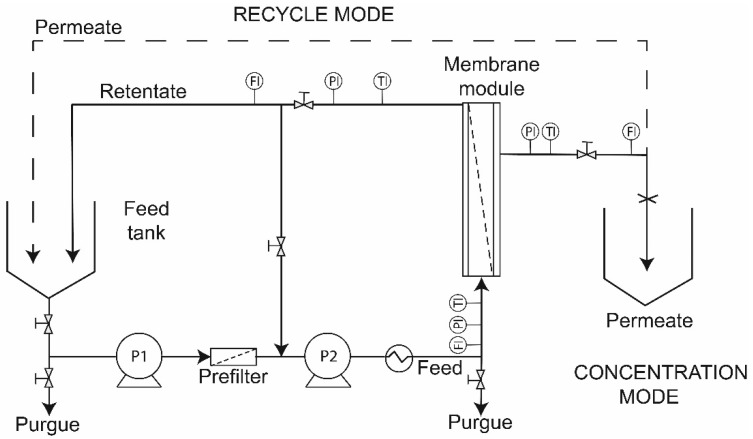
Schematic diagram of the ultrafiltration pilot scale unit used. P1: centrifugal pump; P2: positive displacement pump; TI: Temperature Indicator; PI: Pressure Indicator; FI: Flow Rate Indicator.

A crossflow pilot-scale filtration unit (original design) was used to carry out all experiments (Figure 1). The system is equipped with a 0.15 m^3^ tank, and an interchangeable membrane module to exchange polymeric and ceramic membranes. The feed flow to the membrane was driven by a centrifugal pump and a positive displacement pump (10SV, Gould, Lake Mary, FL, USA) connected in series. The system had flow, pressure and temperature sensors connected to a programmable logic controller (PLC) with a digital panel display to monitor and control the operational parameters. To reach the operating temperature, a heat exchanger placed before the membrane module was used. The operating pressure was manually adjusted with valves in permeate and retentate line streams.

**Figure 2:**
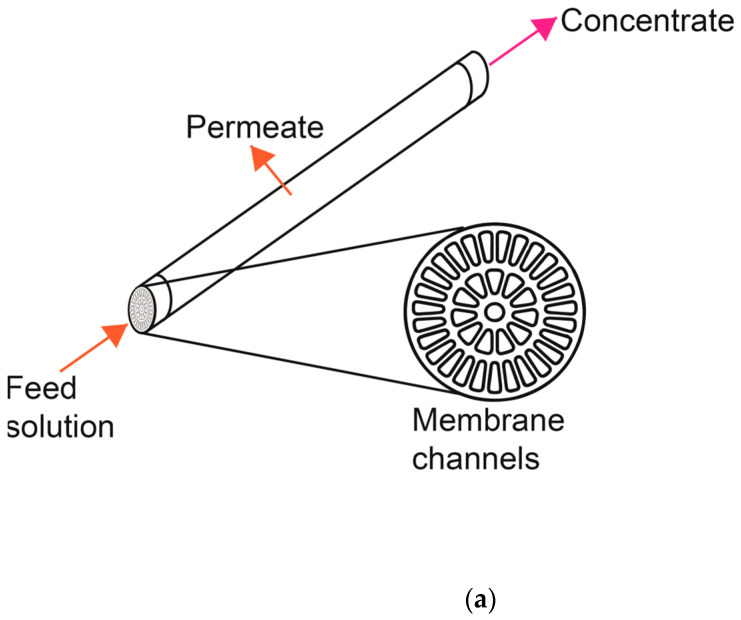

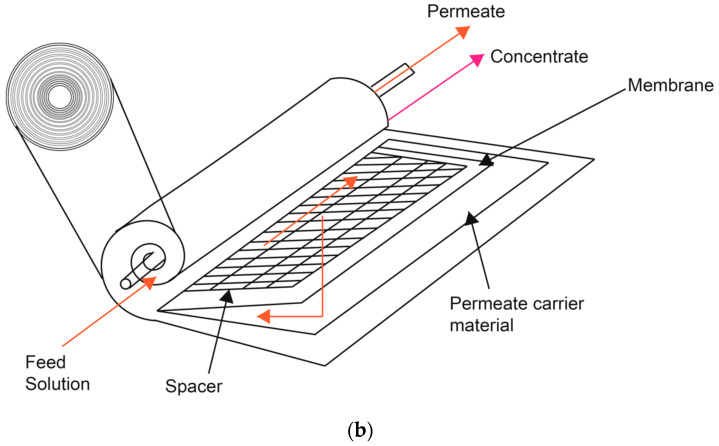
Schematic diagram of tubular ceramic membrane (**a**) and polymeric spiral-wound membrane (**b**).

To compare the ceramic and polymeric membrane systems, new membranes were used for the experiments—a 39-channel ceramic membrane (inside-Céram, TAMI Industries, Les Laurons, Nyons, France) and a spiral polymeric membrane (Hydracore 50, Hydranautics Company, Oceanside, CA, USA) with the same MWCO of 1 kDa (Figure 2). The characteristics of these membranes are shown in Table 1.

### 2.3. Measurement of Size Distribution of Fructans

The size distribution of fructans at initial, retentate and permeate streams was analyzed by HPLC–size exclusion chromatography (SEC), using a 1220 Infinity LC System for HPLC coupled with a refractive index detector (Agilent, Alpharetta, GA, USA) and an Ultrahydrogel DP column and guard column (7.8 mm d.i. × 300 mm, Waters, Milford, MA, USA) in the stationary phase, according to the methodology proposed by Moreno-Vilet et al. (2017) [44]. This technique allowed us to obtain a relative abundance of fructans separated in three groups or fractions for practical purposes: fructans with DP ˃ 10 (Fc), FOS with DP between 3 and 10 and mono-disaccharides (MD) with DP of 1–2, such as glucose, fructose and sucrose.

### 2.4. Evaluation of Membrane Systems Performance

The carbohydrate separation process for each membrane system (ceramic and polymeric) was evaluated in total recycle and concentration modes. The total recycle mode was first carried out in order to compare both membrane systems in terms of permeate flux and selectivity (based on the rejection coefficient). The above means that the permeate and retentate streams of the UF pilot unit were returned to the feed tank at adjusted conditions until reaching a steady state, when the permeate flux and Total Soluble Solids (TSS) did not vary with time; at that moment, the sampling and measurements were carried out. The permeate flux was measured and samples of the permeate and concentrate were collected at 1 × 105, 3 × 105 and 5 × 105 Pa of transmembrane pressure (TMP). The system was operated in batches in concentrations of 10 kg·m^−3^ of aqueous extract because it is the usual concentration of agave juices after extraction in industrial diffusers [45,46]. A temperature of 318 K and a feed flow of 1.8 m^3^·h^−1^ were chosen based on our previous experience and on recommendations by Flores Montaño et al. (2015) [18].

Once the operating conditions were selected, experiments were carried out in concentration mode under the same conditions of temperature, TMP and 0.1 m^3^ feed concentration at 10 kg·m^−3^ for both membrane systems. These experiments permitted us to quantify the overall yield and purification of each carbohydrate fraction, as well as to quantify the fouling phenomenon. The system was operated in batch mode, where the permeate stream was collected in a tank and the retentate stream was recycled to a feed tank. The volume reduction factor (VRF) was calculated as the ratio between the initial feed volume ($V_{f}$) and the retentate volume ($V_{r})$ at any given time (Equation (1)). Samples of the permeate stream were collected for the HPLC–SEC analysis at VRF values of 1.11, 1.25, 1.42, 1.66, 2 and 2.5.

(1) $$VRF=\frac{V_{f}}{V_{r}}$$

#### 2.4.1. Water Permeability and Permeate Flux

The water permeability ($L_{p}$) was measured before and after each experiment to calculate the resistances of the fouling phenomena. First, the membranes were cleaned with demineralized water for about 2–3 h to remove any residual sodium metabisulfite. The pure water permeability was obtained from the slope of the permeate flux as a function of TMP using Equation (2).

(2) $$J_{w}=\frac{TMP}{\mu_{w}\cdot R_{m}}=L_{p}\cdot TMP$$

where $J_{w}$ is the permeate flux with pure water, $\mu_{w}$ is the pure water viscosity and $R_{m}$ the intrinsic membrane resistance [47,48].

#### 2.4.2. Estimation of Rejection Coefficient of Agave Fractions

To estimate the membrane selectivity for each carbohydrate fraction, the solute concentration in permeate stream ($C_{p}$) was related to the feed concentration ($C_{f})$ through the observed rejection coefficient of the membrane by the following equation [49].

(3) $$R_{o}=\left(1-\frac{C_{P,i}}{C_{f,i}}\;\right)\cdot 100\%$$

#### 2.4.3. Analysis of the Fouling Resistance

To quantify membrane fouling during UF performance, different resistances were calculated. The total resistance of the membrane system $R_{t}$ is the sum of the intrinsic membrane resistance $\left(R_{m}\right)$ plus the fouling resistances $\left(R_{f}\right)$, as expressed in Equation (4) [50].

(4) $$R_{t}=\;R_{m}+\;R_{f}$$

Thus, the $R_{m}$ was estimated by solving Equation (2), and $R_{t}$ was also calculated from Equation (2), but considering agave fructan solution data as the experimental permeate flux ($J_{P}$) and the viscosity of the solution in the permeate stream ($\mu_{p}$), which were considered equal to that of water, as it is a dilute solution (<25% w/v) [51,52]. By substituting these considerations into Equation (4), we can obtain the following equation to finally solve $R_{f}$.

(5) $$R_{f}=\frac{TMP}{\mu_{p}\cdot \;J_{P}}-R_{m}$$

where $R_{f}$ represents reversible $\left(R_{ef}\right)$ and irreversible fouling $\left(R_{if}\right)$. Irreversible fouling was estimated by considering the water permeate flux $\left(J_{fw}\right)$ in Equation (6) at the end of the experiment [53].

(6) $$R_{if}=\frac{TMP}{\mu_{w}\cdot \;J_{fw}}-R_{m}$$

Therefore, $R_{ef}$ can be obtained by the difference between the $R_{f}$ value obtained from Equation (5) and the $R_{if}$ value from Equation (6).

#### 2.4.4. Global Yield of the Process

The yield of each fraction of agave fructan (i-solute) was calculated as the total quantity recovered in the permeate or concentrate (M2) in relation to the amount present in the feed (M1), expressed as a percentage:

(7) $$Yield=\left(\frac{M2_{i-Solute}}{M1_{i-Solute}}\right)\cdot 100\%$$

The purification degree of each agave fructan fraction was calculated as the total quantity recovered in the permeate or concentrate (M2) in relation to the total amount present (M2 total), expressed as a percentage:

(8) $$Purity=\;\left(\frac{M2_{i-Solute}}{M2_{Solute}^{Total}}\right)\cdot 100\%$$

#### 2.4.5. Data Analysis

To verify the difference between both systems, a statistical analysis of variance (ANOVA) of permeate flux and the rejection coefficients was performed, using the Statgraphics centurion XVI software. All the experiments were performed in triplicate.

## 3. Results and Discussion

### 3.1. Size Distribution Profile of Agave Fructans

**Figure 3:**
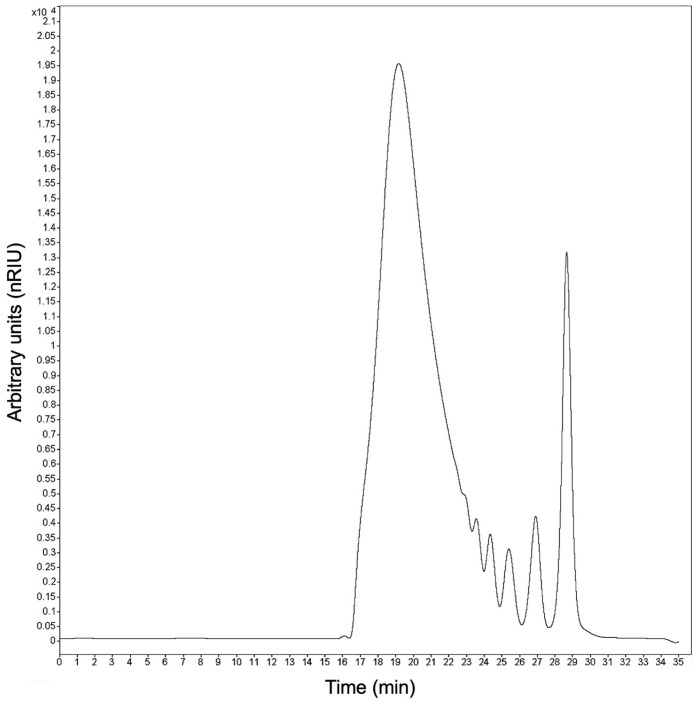
HPLC–size exclusion chromatography (SEC) chromatogram of initial agave fructan sample.

The carbohydrate distribution of commercial agave fructans was quantified by finding 11.33% of MD, 23.77% of FOS and 64.90% of fructans with DP > 10, and a size distribution from one to 42 DP (see Figure 3 and Table 2). These results are consistent with the reports of Moreno-Vilet et al. (2017) [44], where the profile of MW changes in the function of the physiological state of maturity of the plant [54].

### 3.2. Full Recycle Mode Experiments

#### 3.2.1. Water Permeability and Permeate Flux of Systems

**Figure 4:**
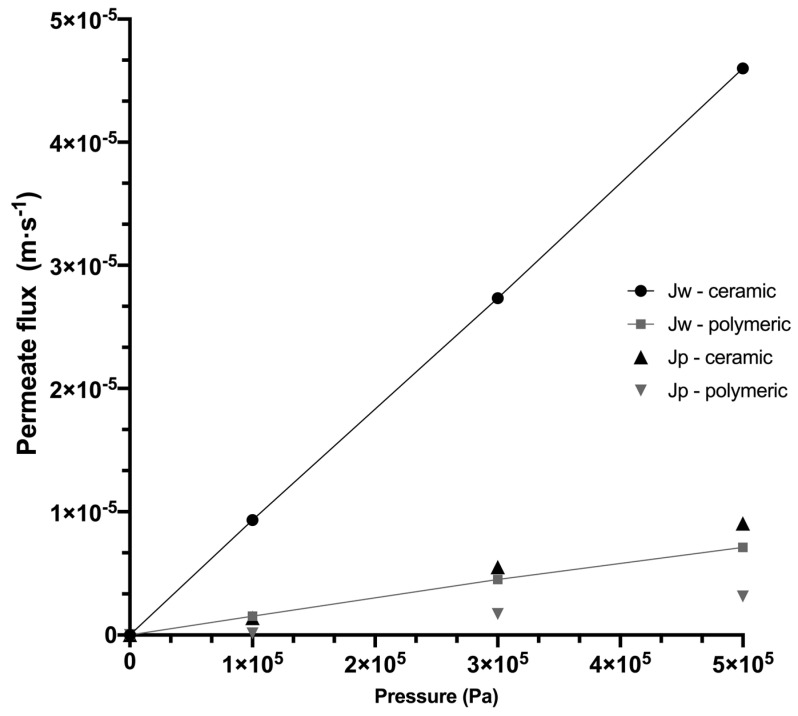
Variation of water flux ($J_{w}$) and fructan permeate fluxes with transmembrane pressure for ceramic and polymeric membrane systems, at feed flow of 1.8 m^3^·h^−1^ and 318.15 K.

To quantify membrane fouling during membrane filtration, it is necessary to know the reference permeate flux by using pure water. This also allows us to guarantee the repeatability of the experiments and the integrity of the membrane. Figure 4 presents the permeate flux as a function of TMP obtained for ceramic and polymeric membrane systems, where the slope that intercepts equal to zero, according to Equation (2), represents the hydraulic permeability. Thus, greatly different hydraulic permeabilities of $L_{p}$= 9.16 × 10^−11^ m^2^·s·kg^−1^ and $L_{p}$= 1.42 × 10^−11^ m^2^·s·kg^−1^ for ceramic and polymeric membrane systems were obtained, respectively. In this sense, for the ceramic membrane, the high value of pure water flux $J_{w}$ at adjusted experimental conditions is also observed, which is attributed to the high estimated tangential velocity of 3 m·s^−1^ and high Reynolds number (see Table 3), characteristic of a turbulent flow pattern (Re>3000). In contrast, the polymeric membrane system reached a low pure water flux $J_{w}$ with a tangential velocity of 0.16 m·s^−1^ and a Reynolds number characteristic of a laminar flow regimen (Re<2000). These results show the different hydrodynamic conditions reached in each system, despite being membranes of the same MWCO and the same feed flow; these differences can be properly attributed to the configurations of membranes (tubular and spiral-wound), which will have important repercussions, both for the selectivity of solutes and for concentration polarization and fouling.

During the UF of agave fructan solution, the evolution of the permeate fluxes with TMP follows a behavior similar to that obtained with pure water, but at a lower flux scale, where the differences between both systems are less radical, but maintain a greater flux in the ceramic membrane. The flux drop between pure water and agave solutions was due to the presence of solutes that involve concentration polarization phenomena in the boundary layer of the membrane. Similar results were found by Grangeon and Lescoche (2000) [40] when comparing tubular ceramic membranes with ceramic flat membranes under identical conditions of temperature, TMP and MWCO, where the highest permeate flux was obtained with the tubular membranes, resulting from the highest tangential velocity used. This is also consistent with Cheryan (1998) [55], who reports tangential velocity values of 7 m·s^−1^ for ceramic tubular membranes and 1 m·s^−1^ for polymeric membranes.

#### 3.2.2. Rejection Coefficients

**Figure 5:**
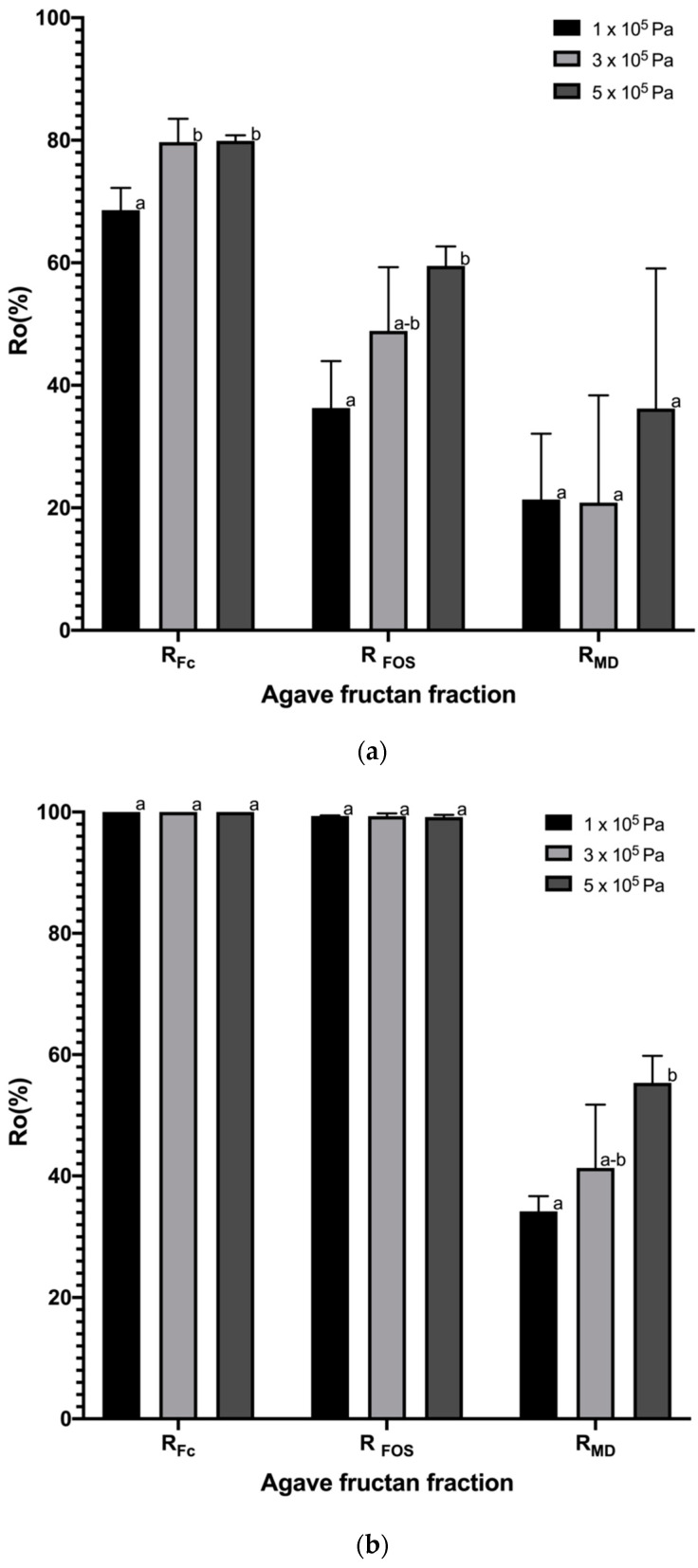
Observed rejection coefficient of agave fructan fraction: fructans with DP ˃ 10 (Fc), fructooligosaccharides (FOS), mono-disaccharides (MD), using a ceramic membrane system (**a**) and polymeric membrane system (**b**) as functions of applied transmembrane pressure. ^a-b^ Different superscripts within the same column indicate that the means differ significantly (*p* ˂ 0.05).

In order to compare the membrane selectivity of both systems (ceramic and polymeric), the rejection coefficient was calculated in three different fractions, grouped by size as fructans with DP > 10 (MW: 1801.56–5000 kg·kmol^−1^), FOS (MW: 504–1639 kg·kmol^−1^) and MD (MW: 180–342 kg·kmol^−1^). Figure 5 shows the observed rejection coefficient of agave fructan fractions for ceramic and polymeric membrane systems, depending on the TMP. For the ceramic membrane system, the rejection values vary, as expected, according to the solute size, as shown in Figure 5a; in general, the rejection of the fractions increased as the TMP increased; this can be attributed to the reduction in pore size caused by the increase in pressure. The lowest rejection coefficients were obtained at the lower TMP of 1 × 10^5^ Pa with values of R_FC_ = 68.58 ± 3.63%, R_FOS_ = 36.29 ± 7.66% and R_MD_ = 21.35 ± 10.75%. The ceramic membrane would be expected to be efficient in the fractionation process since it retains two times more fructans DP > 10 than FOS. These results differ from other studies carried out in the purification process of xylooligosaccharides from liquors of eucalyptus wood and rice husks using the ceramic membrane system [52,56], which reported rejection values of 70 to 93%, which are higher than those found in this work for oligosaccharides; however, it is important to consider that a lot of components are generated during hydrolysis processes such as monosaccharides, acetic acid, oligomers and acetyl groups linked to oligosaccharides, which can interfere with the separation and contribute to the membrane fouling and therefore the greater rejection of solutes.

For polymeric membrane systems, bigger rejection coefficients were obtained and are represented in Figure 5b, with values of R_FC_ = 100 ± 0.00%, R_FOS_ > 99.33 ± 0.10% and R_MD_ > 34.22 ± 2.45%. It is important to note that the polymeric membrane does not have a significant difference between fructans DP > 10 and FOS rejections, so this would not allow us to carry out an efficient fractionation process. On the other hand, the high rejection found for fructans (considering both fractions), compared to the low rejection of mono- and disaccharides, would make an efficient fructan purification process from this polymeric membrane system. Usually, high rejection values of fructans (considering both fractions, DP ˃ 10 + FOS) > 90% have been reported in the literature for polymeric membrane systems [15,16], which is in agreement with the results reported here. Kuhn et al. (2010) [51] report a lower rejection value of 64% using a spiral polymeric membrane of the same MWCO in the purification of FOS obtained by synthesis. However, this difference can be attributed to the interference produced by fructans with DP ˃ 10 present in agave fructans as a natural source, but not in synthesized ones that avoid the free passage of the middle fractions through the membrane.

Agave fructans are considered neutral charge molecules, so differences in the rejection coefficient can be attributed to the sieving effect, which, in turn, can be directly related to the pore size of the membrane. Since both membranes have the same commercial MWCO of 1 kDa, they were expected to have the same pore size. For ceramic membranes of 1 kDa, an average pore size of 3 nm has been estimated [42], while, for a polymeric membrane similar to that used in this study, a polydisperse distribution, in the range from 0.99 to 3.78 nm, has been reported, presenting 50% of pores with a smaller diameter between 0.99 and 2.12 nm [43]. The above suggests that the ceramic membrane has a larger pore size than the polymeric one, which can explain the lower rejection coefficient values found.

### 3.3. Experiments in Concentration Mode

#### 3.3.1. Solute Flux

**Figure 6:**
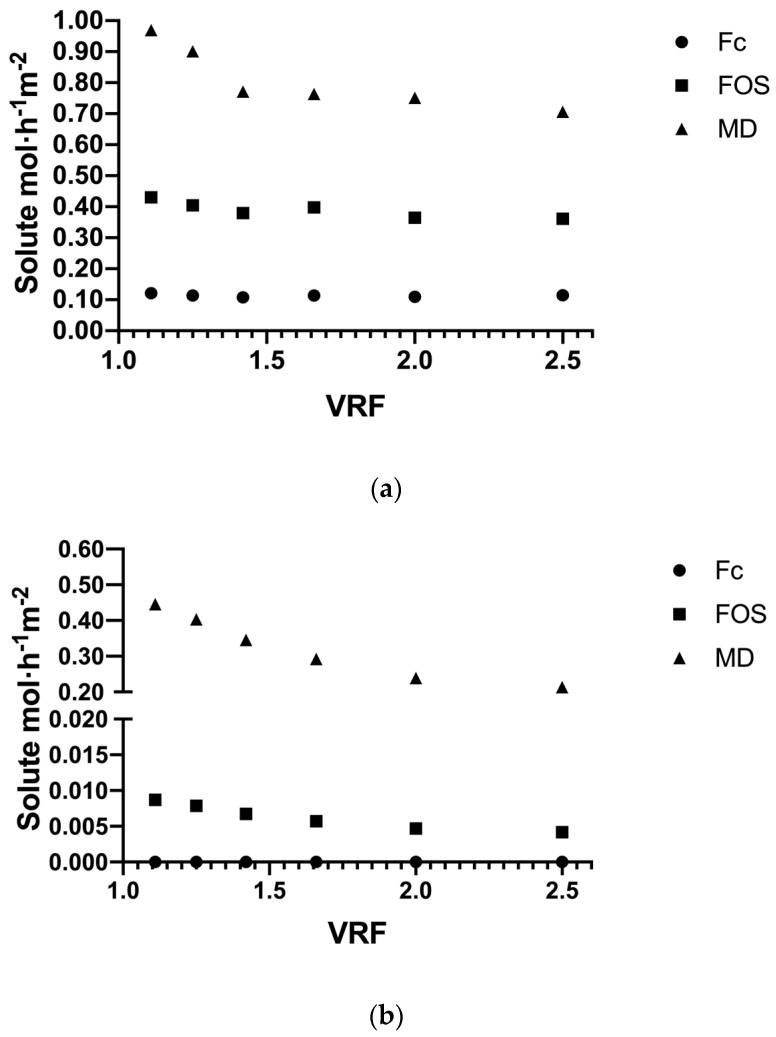
Time evolution as function of volume reduction factor (VRF) of the permeate flux of different solutes during the tight ultrafiltration process for ceramic membrane (**a**) and polymeric membrane (**b**).

The experiments in concentration mode were carried out at 318 K, 3 × 105 Pa of TMP and a 0.1 m^3^ feed concentration at 10 kg·m^−3^ for both membrane systems. Figure 6 shows the results of the different solute fluxes in the permeate stream during UF performance at different VRF values, where the greatest difference is observed in the flux of major solutes such as Fc and FOS between both systems. As expected, the flux of solute moles was determined according to the molecular weight or size of the molecules, where MD had the highest flux, followed by FOS and, finally, the high-molecular weight fructans, Fc, in both systems. The solute flux in the ceramic membrane (Figure 6a) showed a small decrease in all fraction solutes up to achieving VRF at around 1.42, which is attributed to the concentration polarization and initial pore blocking in the membrane. However, the slopes of permeate curves tend toward a constant around 0.75, 0.37 and 0.1 solute mol·h^−1^·m^−2^, for MD, FOS and Fc, respectively, from a VRF of 1.42 to the end of the performance. For the polymeric membrane system (Figure 6b), there was no presence of Fc in the permeate, since 100% is retained by the membrane and the FOS flux (< 0.01 solute mol·h^−1^·m^−2^) was 45 times less compared to the ceramic system. The MD flux in the polymeric system showed a progressive decrease during the UF performance starting at 0.56 and ending at 0.2 solute mol·h^−1^·m^−2^. These results show how the hydrodynamic conditions in the systems mainly affect the larger molecules (Fc and FOS), forcing their passage through the ceramic membrane (turbulent flow), but not in the polymeric membrane (laminar flow), while mono- and disaccharides are the smallest solutes with free passage through different membranes.

#### 3.3.2. Analysis of the Fouling Resistance

**Figure 7:**
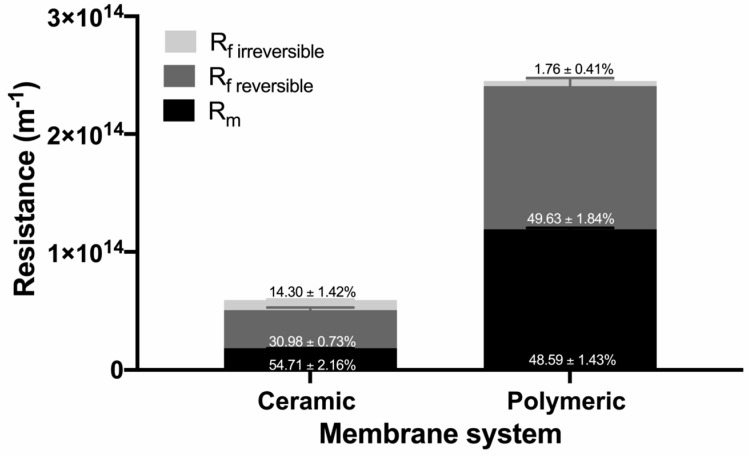
Calculated resistances of the membrane systems—reversible and irreversible fouling.

One of the main drawbacks of membrane technology is the decrease in permeate flux, resulting from the fouling of the membrane, which implies process stoppages and regular cleaning of the membranes. This can be attributed to concentration polarization phenomena and the increase in additional resistance generated by the molecules on the membrane surface and/or pore blocking. Figure 7 shows the calculated values of the resistance from experiments carried out using either ceramic or polymeric membrane systems obtained for a concentration mode when 0.1 m^3^ solution was treated. For the ceramic membrane system, the intrinsic membrane resistance $R_{m}$ contributes 54.71 ± 2.16% of the total resistance, so 30.98 ± 0.73% corresponds to the reversible fouling ($R_{ef})$, which means that it can be removed using backwash with water. On the other hand, only 14.30 ± 1.42% of the fouling corresponds to irreversible fouling, which suggests that few subsequent washes with chemical agents are required to recover the reference flow. In contrast, the polymeric membrane system presents an $R_{m}$ value that corresponds to 48.59 ± 1.43% of the total resistance during the experiment with agave fructans, while 49.63 ± 1.84% corresponds to reversible fouling ($R_{ef})$, meaning that it can be easily removed with rinses, and the weak value of irreversible fouling requires less use of chemicals during the cleaning process, increasing the operational lifetime expectancy of the polymeric membrane.

Concentration polarization involves the accumulation of solutes in the boundary layer of the membrane that could produce fouling. In this sense, as mentioned before with the Reynolds number in Table 3, a turbulent flow is achieved, using high tangential velocities, which result in a thinner boundary layer and less reversible fouling; therefore, the ceramic membrane is favored over the polymeric membrane in this sense. On the other hand, the molecules could be trapped inside, blocking the pores of the ceramic membrane due to its larger pore size [57], favoring irreversible fouling. In the case of the polymeric membrane, the high percentage of reversible fouling can be explained by the low Re values used in the system (laminar flow), which favor the deposition of particles on the surface of the membrane acting as an additional barrier and favoring solute rejection.

#### 3.3.3. Analysis of Global Process

**Figure 8:**
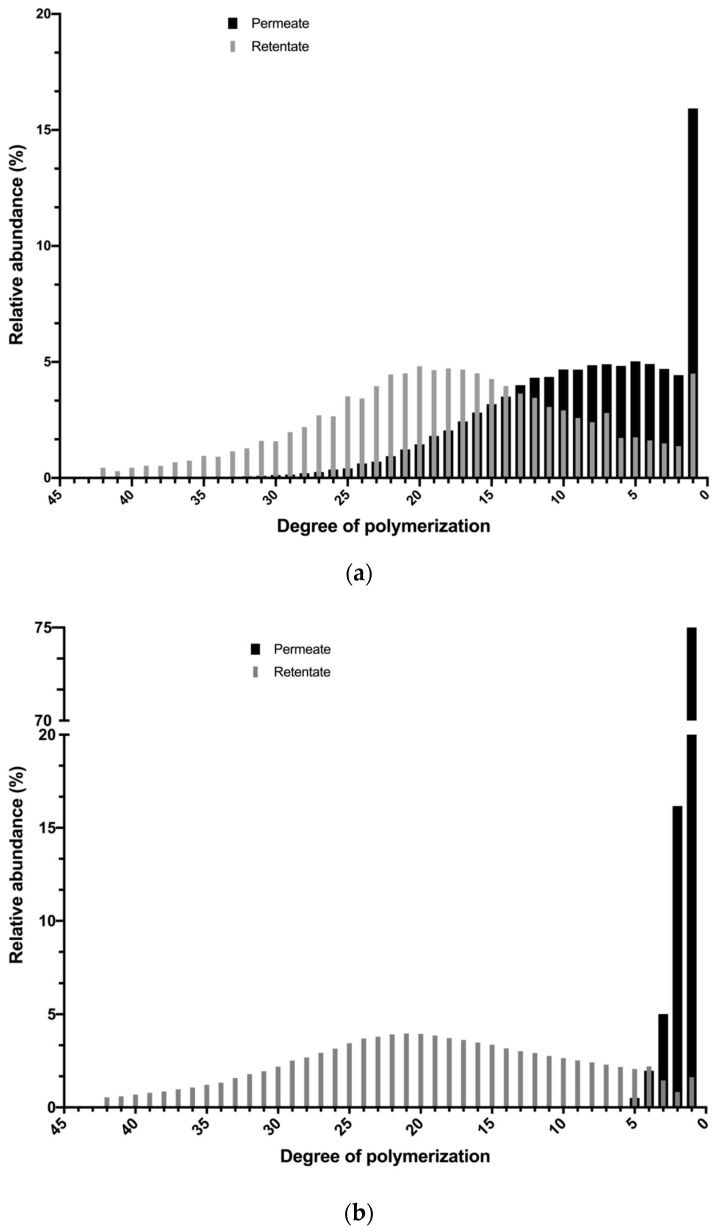
Fructan size distribution histograms of the final retentate and permeate solutions obtained with ceramic (**a**) and polymeric (**b**) membrane systems for a batch process.

Figure 8 shows the final fructan composition of retentate and permeate in size distribution histograms for both systems. The yield and purity of Fc, FOS, and MD in the retentate and permeate at the end of the total process (concentration mode) were affected according to the membrane system used and the results are reported in Table 4.

For the ceramic membrane system (Figure 8a), a wider distribution of fructans in the permeate is obtained with DP in the range of 1–32 and an average DP of 9.1. It means that the FOS fraction was concentrated in a permeate stream with a purity of 42.16% and a yield of 40.06%, as well as the 50.02% of MD fraction with 20.78% of purity, while the Fc fraction reached the highest purity (78.23%) in the retentate with a yield of 70.91%, with an average DP of 17.9. These results confirm the potential use of the ceramic membrane system for the fractionation of agave fructans and to obtain a product enriched in FOS in the permeate and a product rich in Fc in the retentate. The FOS obtained can be incorporated into the production of food with a prebiotic effect and the formulation of supplements, while the Fc can be used for its technological properties.

For a polymeric membrane system, the cut was more drastic, where the permeate was obtained with a very short distribution of fructans with DP between one and five and average DP of 1.34 (see Figure 8b). This means that the 33% MD fraction was recovered from the permeate with a 94% purity, while 89% of Fc and 45% of FOS fractions were recovered in the retentate with a purity of 82% and 15%, respectively, which led to an average DP of 20.69. The low yields obtained from FOS and MD in the polymeric system can be attributed to the deposition of these solutes on the membrane, which can be translated into the large reversible fouling values that this system presented. Experimentally, these solutes were recovered by rinsing the system. These results confirm the potential use of polymeric membranes to obtain a purified product of agave fructans (Fc + FOS) in the retentate with a purity of 97.74% and yield of 64.28%, which allows us to improve the techno-functional properties of agave fructans and reduce the difficulties of the drying process associated with the thermoplastic characteristics of low-molecular weight sugars.

Finally, it is important to note that the process times in the concentration mode were 300 min and 33 min for the ceramic and polymeric systems, respectively, which were the result of the area of each membrane used.

## 4. Conclusions

Great performance differences were found in our comparison of ceramic and polymeric membrane systems for the tight UF of agave fructans. The hydraulic permeability, permeate flux and rejection coefficient were affected by the membrane type and TMP, using the same temperature, feed concentration and MWCO of membranes for both systems, where the ceramic membrane system presented higher hydraulic permeability and permeate flux, but also greater irreversible fouling compared to the polymeric membrane. The hydrodynamic conditions defined by the configuration and material of the membrane, independently of MWCO, largely define the pattern of solute separation and fouling during a performance, so they are important conditions to consider in future works. The global yield of the process results in very different permeation patterns between both systems at the same operational conditions. Therefore, the membranes studied can be used for different purposes; the ceramic membrane system could be used to fractionate agave fructans and thus obtain products with different MW profiles, while the polymeric membrane system could be used for purification, meaning mono- and disaccharide-free agave fructans.

## Figures and Tables

**Table 1:** Characteristics of tight ultrafiltration membranes used.

Specification	Ceramic Membrane	Polymeric Membrane
Manufacturer	Tami industries	Hydranautics Nitto Group Company
Material	Zirconium/titanium dioxide	Sulfonated polyethersulfone
Configuration	Tubular	Spiral
Molecular weight cut-off	1 kDa	1 kDa
Membrane area	0.5 m^2^	7.4 m^2^
Operating pressure	<100 × 10^5^ Pa	<41 × 10^5^ Pa
Operating pH	0–14	2–11
Operating temperature	<350 °C	<50 °C
Spacer height *	NA	8.63 × 10^−4^ m *
Spacer porosity *	NA	0.89 *
Mean pore radio (nm)	3 **	2.12 ***

* [41], ** [42], *** [43].

**Table 2:** Profile of polymerization and mass distribution of commercial agave fructan, obtained with HPLC–SEC analysis.

DP	Name	Formula	MW kg·kmol^−1^	%
**1**	Glucose and Fructose	C_6_H_12_O_6_	180	8.60
**2**	Sucrose	C_12_H_22_O_11_	342	2.73
**3**	Fructose (Fructose)_1_ Glucose	C_18_H_32_O_16_	504	2.30
**4**	Fructose (Fructose)_2_ Glucose	C_24_H_42_O_21_	666	2.43
**5**	Fructose (Fructose)_3_ Glucose	C_30_H_52_O_26_	828	2.66
**6**	Fructose (Fructose)_4_ Glucose	C_36_H_62_O_31_	990	2.65
**7**	Fructose (Fructose)_5_ Glucose	C_42_H_72_O_36_	1152	4.31
**8**	Fructose (Fructose)_6_ Glucose	C_48_H_82_O_41_	1314	3.03
**9**	Fructose (Fructose)_7_ Glucose	C_54_H_92_O_46_	1476	3.09
**10**	Fructose (Fructose)_8_ Glucose	C_60_H_102_O_51_	1638	3.29
**11**	Fructose (Fructose)_9_ Glucose	C_66_H_112_O_56_	1800	3.30
**12**	Fructose (Fructose)_10_ Glucose	C_72_H_122_O_61_	1962	3.54
**13**	Fructose (Fructose)_11_ Glucose	C_78_H_132_O_66_	2124	3.58
**14**	Fructose (Fructose)_12_ Glucose	C_84_H_142_O_71_	2286	3.75
**15**	Fructose (Fructose)_13_ Glucose	C_90_H_152_O_76_	2448	3.88
**16**	Fructose (Fructose)_14_ Glucose	C_96_H_162_O_81_	2610	3.95
**17**	Fructose (Fructose)_15_ Glucose	C_102_H_172_O_86_	2772	3.96
**18**	Fructose (Fructose)_16_ Glucose	C_108_H_182_O_91_	2934	3.88
**19**	Fructose (Fructose)_17_ Glucose	C_114_H_192_O_96_	3096	3.71
**20**	Fructose (Fructose)_18_ Glucose	C_120_H_202_O_101_	3258	3.74
**21**	Fructose (Fructose)_19_ Glucose	C_126_H_212_O_106_	3420	3.41
**22**	Fructose (Fructose)_20_ Glucose	C_132_H_222_O_111_	3582	3.29
**23**	Fructose (Fructose)_21_ Glucose	C_138_H_232_O_116_	3744	2.85
**24**	Fructose (Fructose)_22_ Glucose	C_144_H_242_O_121_	3906	2.42
**25**	Fructose (Fructose)_23_ Glucose	C_150_H_252_O_126_	4068	2.43
**26**	Fructose (Fructose)_24_ Glucose	C_156_H_262_O_131_	4230	1.80
**27**	Fructose (Fructose)_25_ Glucose	C_162_H_272_O_136_	4392	1.80
**28**	Fructose (Fructose)_26_ Glucose	C_168_H_282_O_141_	4554	1.44
**29**	Fructose (Fructose)_27_ Glucose	C_174_H_292_O_146_	4716	1.28
**30**	Fructose (Fructose)_28_ Glucose	C_180_H_302_O_151_	4878	1.01
**31**	Fructose (Fructose)_29_ Glucose	C_186_H_312_O_156_	5040	1.01
**32**	Fructose (Fructose)_30_ Glucose	C_192_H_322_O_161_	5202	0.79
**33**	Fructose (Fructose)_31_ Glucose	C_198_H_332_O_166_	5364	0.71
**34**	Fructose (Fructose)_32_ Glucose	C_204_H_342_O_171_	5526	0.56
**35**	Fructose (Fructose)_33_ Glucose	C_210_H_352_O_176_	5688	0.58
**36**	Fructose (Fructose)_34_ Glucose	C_216_H_362_O_181_	5850	0.46
**37**	Fructose (Fructose)_35_ Glucose	C_222_H_372_O_186_	6012	0.41
**38**	Fructose (Fructose)_36_ Glucose	C_228_H_382_O_191_	6174	0.32
**39**	Fructose (Fructose)_37_ Glucose	C_234_H_392_O_196_	6336	0.32
**40**	Fructose (Fructose)_38_ Glucose	C_240_H_402_O_201_	6498	0.27
**41**	Fructose (Fructose)_39_ Glucose	C_246_H_412_O_206_	6660	0.18
**42**	Fructose (Fructose)_40_ Glucose	C_252_H_422_O_211_	6822	0.29
			Total	100

**Table 3:** Experimental conditions for the tight ultrafiltration process.

Specification	Ceramic Membrane	Polymeric Membrane
Operating pressure (Pa)	3 × 10^5^	3 × 10^5^
Tangential velocity (m·s^−1^)	3	0.16
Reynolds number *	12,460	398.74
Operating temperature (K)	318	318
Feed Concentration (kg·m^−3^)	100	100
Hydraulic diameter (m)	0.0025	0.0015

* Data estimated using a $\rho_{s}$ = 990.22 kg·m^−3^, µ = 596 × 10^−6^ kg·m^−1^·s^−1^, v = 3 m·s^−1^ for the ceramic membrane and v = 0.16 m·s^−1^ for the polymeric membrane.

**Table 4:** Global yield of tight ultrafiltration (UF) process with ceramic and polymeric membrane systems.

	Ceramic Membrane System			Polymeric Membrane System		
	Fc	FOS	MD	Fc	FOS	MD
Feed solution (kg)	6.490	2.377	1.133	6.490	2.377	1.133
Final yield of the permeate (%)	11.05 ± 3.69^a^	40.06 ± 0.70^a^	52.08 ± 1.76^a^	0.00^b^	0.98 ± 0.88^b^	33.22 ± 0.79^b^
Purity of the permeate (%)	37.05 ± 2.73^a^	42.16 ± 2.09^a^	20.78 ± 0.63^a^	0.00^b^	5.87 ± 2.23^b^	94.12 ± 2.23^b^
Final yield of the retentate (%)	70.91 ± 3.11^a^	48.66 ± 9.45^a^	43.72 ± 9.43^a^	89.07 ± 8.00^b^	45.30 ± 2.97^a^	13.90 ± 0.09^b^
Purity of the retentate (%)	78.23 ± 0.73^a^	16.24 ± 0.62^a^	5.52 ± 0.10^a^	82.38 ± 0.48^b^	15.36 ± 0.28^a^	2.25 ± 0.20^b^

^a-b^ Different superscripts within the same column of the same fractions between both systems indicate that the means differ significantly (*p* ˂ 0.05).

## Nomenclature

*Symbols*	
*A*	membrane area (m^2^)
$C_{P,i}$	concentration of solute i in permeate stream (kg·m^−3^)
$C_{f,i}$	concentration of the solute i in feed (kg·m^−3^).
$\mu_{p}$	viscosity of fructans in permeate stream (kg·m^−1^·s^−1^)
$\mu_{w}$	water viscosity (kg·m^−1^·s^−1^)
$\rho_{s}$	density of fructans solution (kg·m^−3^)
Mw	molecular weight (kg·kmol^−1^)
$J_{P}$	permeate flux with fructan solution (m·s^−1^)
$J_{w}$	permeate flux with pure water (m·s^−1^)
$L_{p}$	hydraulic permeability (m^2^·s·kg^−1^)
Re	Reynolds number (dimensionless)
$R_{f}$	fouling resistance (m^−1^)
$R_{t}$	total resistance of the membrane system (m^−1^)
$R_{o}$	observed rejection (dimensionless)
$R_{FC}$	rejection coefficient of agave fructan with DP ˃ 10 (dimensionless)
$R_{FOS}$	rejection coefficient of FOS (dimensionless)
$R_{MD}$	rejection coefficient of mono- and disaccharides (dimensionless)
$R_{m}$	intrinsic membrane resistance (m^−1^)
TMP	transmembrane pressure (Pa)
dh	hydraulic diameter of membrane (m)
v	tangential velocity (m·s^−1^)
$V_{r}$	retentate volume (m^3^)
$V_{f}$	feed volume (m^3^)
